# Methanol bioconversion into C3, C4, and C5 platform chemicals by the yeast *Ogataea polymorpha*

**DOI:** 10.1186/s12934-023-02283-z

**Published:** 2024-01-03

**Authors:** Katrin Wefelmeier, Simone Schmitz, Benjamin Jonas Kösters, Ulf Winfried Liebal, Lars Mathias Blank

**Affiliations:** iAMB - Institute of Applied Microbiology, ABBt – Aachen Biology and Biotechnology, RWTH Aachen University, Worringerweg 1, D-52074 Aachen, Germany

## Abstract

**Background:**

One carbon (C1) molecules such as methanol have the potential to become sustainable feedstocks for biotechnological processes, as they can be derived from CO_2_ and green hydrogen, without the need for arable land. Therefore, we investigated the suitability of the methylotrophic yeast *Ogataea polymorpha* as a potential production organism for platform chemicals derived from methanol. We selected acetone, malate, and isoprene as industrially relevant products to demonstrate the production of compounds with 3, 4, or 5 carbon atoms, respectively.

**Results:**

We successfully engineered *O. polymorpha* for the production of all three molecules and demonstrated their production using methanol as carbon source. We showed that the metabolism of *O. polymorpha* is well suited to produce malate as a product and demonstrated that the introduction of an efficient malate transporter is essential for malate production from methanol. Through optimization of the cultivation conditions in shake flasks, which included pH regulation and constant substrate feeding, we were able to achieve a maximum titer of 13 g/L malate with a production rate of 3.3 g/L/d using methanol as carbon source. We further demonstrated the production of acetone and isoprene as additional heterologous products in *O. polymorpha*, with maximum titers of 13.6 mg/L and 4.4 mg/L, respectively.

**Conclusion:**

These findings highlight how *O. polymorpha* has the potential to be applied as a versatile cell factory and contribute to the limited knowledge on how methylotrophic yeasts can be used for the production of low molecular weight biochemicals from methanol. Thus, this study can serve as a point of reference for future metabolic engineering in *O. polymorpha* and process optimization efforts to boost the production of platform chemicals from renewable C1 carbon sources.

**Supplementary Information:**

The online version contains supplementary material available at 10.1186/s12934-023-02283-z.

## Background

One of the key challenges for society in this century is to mitigate anthropogenic global warming and to establish a sustainable economy within the planetary boundaries [1]. To achieve this, essential platform chemicals and materials must be produced independently of dwindling fossil fuel resources and with minimized greenhouse gas emissions [2]. A bioeconomy, in which fossil feedstocks are replaced with biological substrates could play an essential role in achieving this goal [3]. It has been estimated that producing bulk chemicals using industrial biotechnology rather than petrochemical processes could save 500–1000 million tons of CO_2_ per year globally [4]. Microorganisms, in particular, possess unique properties that enable the production of complex multi-carbon compounds with high efficiency under mild reaction conditions. However, most microbial production processes currently rely on glucose as a carbon source, which requires arable land and thus potentially competes with the food sector [5]. This is particularly problematic if increasing quantities of sugars were to be used for producing large quantities of bulk chemicals. An alternative to glucose feedstocks could be the use of one carbon (C1) molecules, (e.g., methane, methanol, or formic acid). In the future, these molecules could be derived from the hydrogenation of CO_2_ with green hydrogen, providing a means to produce important platform chemicals in a carbon-neutral manner [6, 7].

Several microbes are natively able to use C1 compounds as their sole carbon and energy source, e.g., methylotrophic yeasts. This group of yeasts can assimilate methanol (MeOH) into formaldehyde via the methanol oxidase (MOX) [8]. The produced formaldehyde can then either be oxidized to CO_2_ for energy generation or assimilated to form biomass constituents via the xylulose monophosphate (XuMP) pathway [9]. Using methanol as a carbon source has the advantage that it can be easily stored in its liquid form, which circumvents mass transfer problems that occur during gas fermentations based on CO_2_ or methane [10]. A promising methylotrophic yeast is *Ogataea polymorpha.* It is known for its high protein secretion rates [11, 12] and thus has been used to produce heterologous proteins on an industrial scale [13]. Apart from that, its thermotolerance and strong methanol-inducible promoters also make it a promising host organism for producing platform chemicals derived from C1 molecules [14, 15]. There are several recent studies, in which industrially relevant chemicals, such as free fatty acids [16], hyaluronic acid [17], and the terpenoid β-elemene [18] were produced in *O. polymorpha*.

**Fig. 1:**
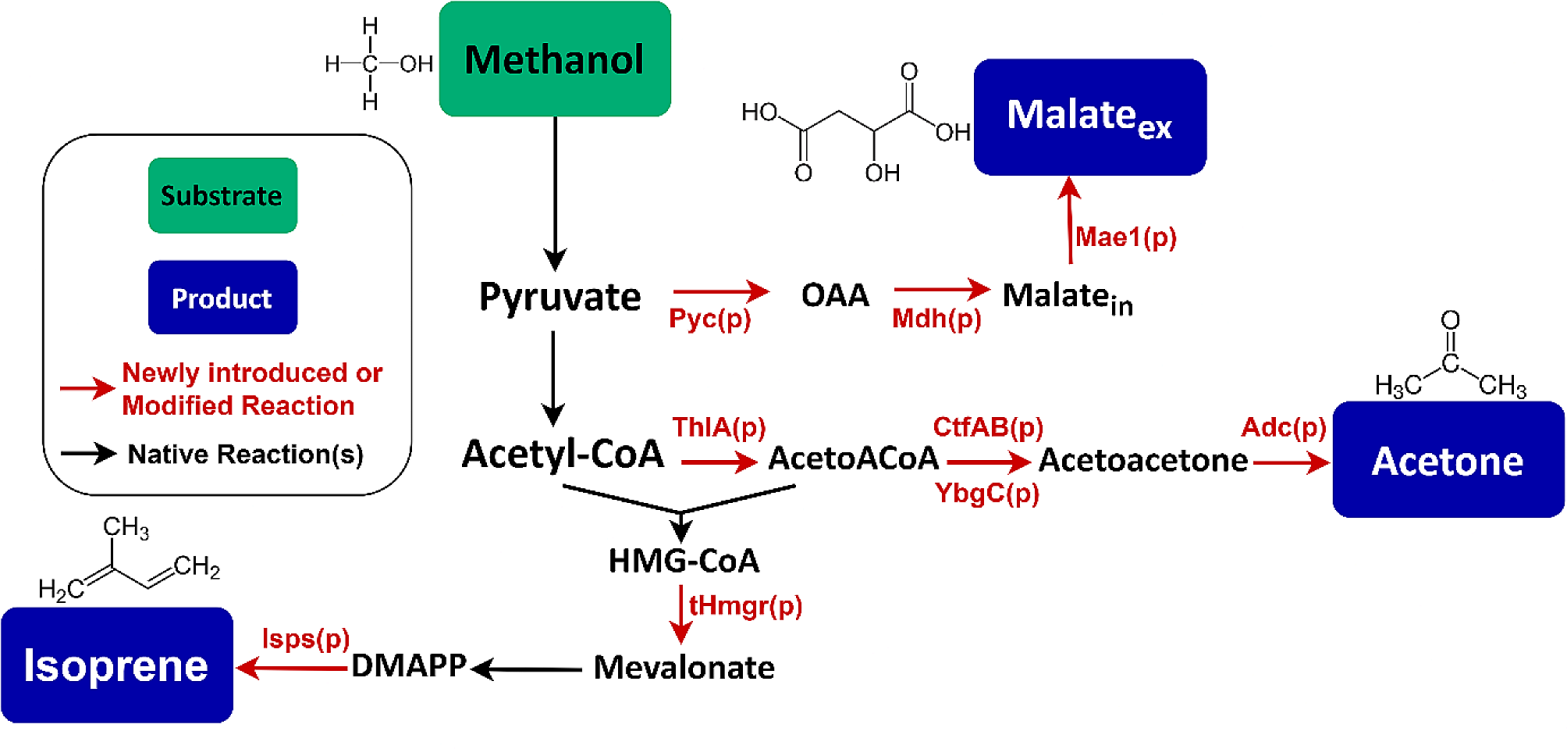
Metabolic modifications introduced into *O. polymorpha* for producing malate, acetone, or isoprene from methanol as carbon source. Used abbreviations: Pyc(P): pyruvate carboxylase, OAA: oxaloacetate Mdh(p): malate dehydrogenase, Mae1(p): malic acid transporter, ThlA(p): acetyl-CoA acetyltransferase, AcetoACoA: acetoacetyl-CoA, CtfAB(p): butyrate-acetoacetate CoA-transferase, YbgC(p): thioesterase, Adc(p): acetoacetate decarboxylase, tHmgr(p): truncated 3-hydroxy-3-methylglutaryl-CoA reductase, Isps(p): isoprene synthase, HMG-CoA: 3-hydroxy-3-methylglutaryl coenzyme A, DMAPP: dimethylallyl pyrophosphate

In this study, we further want to evaluate the potential of *O. polymorpha* to become a versatile cell factory for the production of low molecular weight biochemicals. We modified *O. polymorpha* to produce acetone, isoprene, and malate, as examples of C3-C5 molecules derived from methanol (Fig. 1). The dicarboxylic acid malic acid (C_4_H_6_O_5_) is a main flavor component of many fruits and is thus widely used as a flavor enhancer and acidulant in the food industry [19, 20]. Additionally, it has a broad range of applications in the cosmetics and pharmaceutical industry, but it can also be applied for metal or textile finishing [21, 22]. Further, its polymer poly(malic acid) has many interesting properties, such as its high water solubility, biodegradability, and biocompatibility, which make it an interesting molecule, e.g., for drug delivery systems [23]. Acetone (C_3_H_6_O) is used as a solvent and as an intermediate for polymethyl methacrylate plastics [24]. At the beginning of the 20th century, acetone was already being produced biotechnologically with the bacterium *Clostridium acetobutylicum* in the famous acetone-butanol-ethanol (ABE) fermentation [25]. Today, acetone is produced almost exclusively from fossil resources. The same applies to isoprene (C_5_H_8_), whose main application is in its polymerized form as a rubber material [26, 27]. Also, for isoprene, an alternative microbial production is possible, since there are a large number of isoprene-emitting plants whose isoprene synthases can be used for heterologous expression in microorganisms [28].

As all of these chemicals are needed in large quantities, manufacturing these bulk chemicals using renewable resources, such as C1 molecules, has the potential to considerably reduce the environmental impact of their production. Therefore, this study aims to evaluate the suitability of *O. polymorpha* as a production organism for malate, acetone, and isoprene as proof-of-concept platform chemicals.

## Results & discussion

### Metabolic modelling

**Fig. 2:**
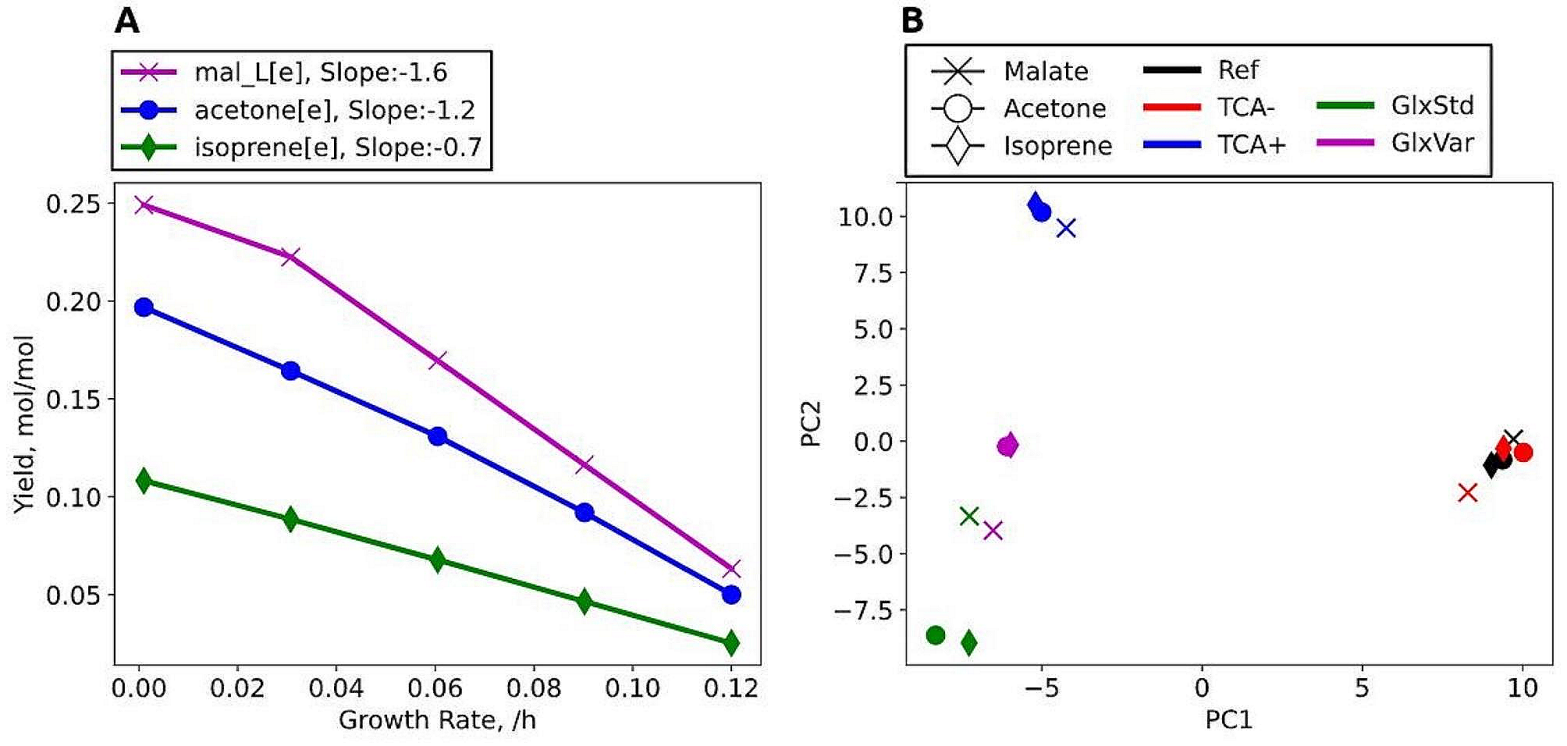
**(A)** Production envelope of the yield for malate, acetone, and isoprene in response to growth rate. A higher slope of malate indicates stronger consumption with growth. **(B)** Principal component analysis of simulated flux distributions of five different metabolic scenarios while optimizing for target production: unconstraint reference flux (Ref: black), inhibition of TCA cycle (TCA-: red), enforcing high TCA cycle (TCA+: blue), enforcing glyoxylate shunt (GlxStd: green) and glyoxylate shunt without malate dehydrogenase (GlxVar: magenta). While the absolute position in the plot is irrelevant, the relative distance of points reflects the degree of similarity between flux distributions. Details about the reactions constrained for each scenario can be found in the SI: Figure S1

The genome scale model (GSMM) of *O. polymorpha* can describe metabolic conditions of malate, acetone, and isoprene production. We used the existing genome scale model iUL909 [29] and added reactions for acetone and isoprene production to generate the updated GSMM iOpol23. The added reactions are isoprene synthase, acetoacetyl-CoA hydrolase, acetoacetate decarboxylase as well as transport reactions. The production envelope in Fig. 2 shows the yield benefits with lower growth rates. Malate has the highest slope, which signifies the importance for the growth metabolism and suggests that growth rate reduction or two-phase production are suitable optimization strategies. It can further be seen that the metabolic pathway for malate production seems to be the most efficient of the three selected products, as with minimal growth almost all the carbon can be directed to product generation, which corresponds to the maximum theoretical yield of 0.25 mol_Malate_/mol_MeOH_.

We tested how rerouting of carbon fluxes to distinct pathways affects the simulated production rates. Besides the unconstraint reference flux (Ref), we investigated the pathway scenarios (I) Blocked TCA (TCA-) with mutations to impair TCA flux (see Methods), (II) Forced TCA activity (TCA+), (III) Forced glyoxylate cycle (GlxStd), and (IV) Forced impaired glyoxylate shunt (GlxVar) with deactivated mitochondrial malate dehydrogenase. The optimal target production route via the Ref-flux is similar to the inactive TCA scenario, because of the comparable yields (Table 1). The yields drop if fluxes are forced via glyoxylate shunt, but this decrease is weaker for acetone and isoprene than for malate. A principal component analysis (PCA) of the flux distributions visualizes the similarity among the solutions (Fig. 2B, Supplementary Excel Sheet 1). Overall, there are three distinct clusters that represent different pathway modes (I) Ref and TCA-, (II) TCA+, and (III) glyoxylate shunt fluxes. The fluxes for the different products are similar within the pathway clusters. Particularly clustered are fluxes of the reference solution (Ref) and inactive TCA (TCA-) and reflect their similarity. Acetone and isoprene (circle/diamond) have a high flux similarity in the different scenarios.

**Table 1:** Simulated yields of optimal target production for different scenarios of metabolic activity. The yield is calculated as the production rate of the respective product divided by the methanol uptake uptake rate (both mmol/gDW/h). ‘Ref’ refers to a simulation without special constraints, ‘TCA-’ was constrained to inhibit circular TCA cycle flux, in ‘TCA+’ flux was forced to enter circular TCA cycle, ‘GlxStd’ represents a flux distribution forced into the standard glyoxylate shunt, and ‘GlxVar’ represents a flux distribution with forced fluxes without malate dehydrogenase activity. The identity of the reactions within the reaction network ist sketched in the SI the Figure S1

Scenario	Ref	TCA-	TCA+	GlxStd	GlxVar
Yield, mol_Malate_/mol_MeOH_	0.25	0.25	0	0.02	0
Yield, mol_Aceton_/mol_MeOH_	0.2	0.19	0	0.02	0
Yield, mol_Isoprene_/mol_MeOH_	0.11	0.10	0	0.01	0

### Malate production

**Fig. 3:**
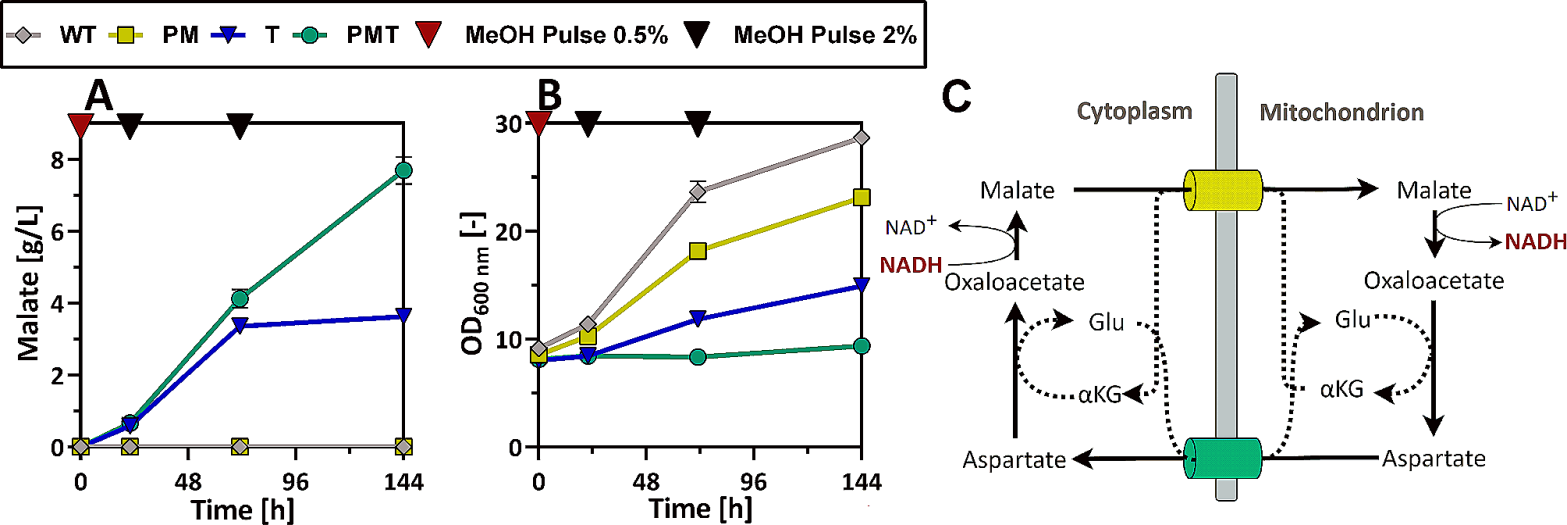
Production of malate (**A**) from methanol and development of optical density (**B**) in *O. polymorpha* cultures. Comparison of the unmodified wildtype (WT) strain, a strain overexpressing the PYC and MDH gene of Rhizopus oryzae (PM), a strain overexpressing the malate transporter gene MAE1 of Schizosaccharomyces pombe (T) and a strain overexpressing all three of these heterologous genes (PMT). Cultivations were performed on Verduyn medium in 250 mL shake flasks. Error bars represent the standard deviation of biological triplicates. (**C**) Illustration of the malate-aspartate shuttle, Used abbreviations: Glutamate (Glu), α-ketoglutarate (α-KG)

As less flux through the TCA cycle seems to be beneficial for malate production from methanol in *O. polymorpha* (cp. Figure 2; Table 1), the reductive TCA cycle (rTCA) as an alternative production pathway was overexpressed in the cytosol of *O. polymorpha* (Fig. 1). The pyruvate carboxylase (Pyc(p), EC:6.4.1.1.) and malate dehydrogenase (Mdh(p), EC:1.1.1.37) from *Rhizopus oryzae* were selected as they have already been successfully used in a number of studies for malate production in yeasts [30, 31]. Additionally, the malic acid transporter Mae1(p) from *Schizosaccharomyces pombe* was introduced as the transportation of malic acid across the plasma membrane has often been described to be a limiting factor in malate production [32, 33]. Mae1(p) was characterized as a voltage-dependent anion channel, and in a comparison of 7 different carboxylic acid transporters, it showed the highest activity toward malate and the least detrimental effects on growth when overexpressed in *Saccharomyces cerevisiae* [34]. All three genes were codon-optimized for *O. polymorpha* and expressed using methanol-inducible promoters (Details on genetic constructs in Supplementary Information (SI): Table S1). To determine the impact of the individual genes on production, strains were engineered that had only the *MAE1* transporter gene (T-strain) or the *MDH* and *PYC* gene (PM-strain) integrated into their genome, in addition to the strain with all three heterologous genes (PMT-strain). The constructed strains were then analyzed for malate production using methanol as the sole carbon source. To this end, pre-cultures were first grown on YPD-medium with glucose (cf. Methods section), in order to obtain a certain amount of biomass. Since all introduced heterologous genes are under the control of methanol-inducible promoters, no malate production takes place during this preculture phase. To induce production, the cells were then washed and transferred to mineral Verduyn medium (cf. SI: Table S2-4) containing only methanol as a carbon source. Figure 3A & B show the malate production and growth behavior on methanol of the engineered strains and the unmodified WT strain. The initial methanol concentration in the Verduyn medium was 4 g/L (0.5%(v/v)) and during cultivation the cells were provided with two additional pulses of 16 g/L (2%) methanol once the methanol concentration was depleted (methanol consumption data in SI: Figure S2). This cultivation method was chosen because our previous studies showed that even low concentrations of methanol are toxic to carboxylic acid-producing *O. polymorpha* strains. The addition of methanol in multiple pulses to the shake flasks was shown to be an effective strategy for reducing methanol toxicity to the cells [35].

While for the WT- and PM-strain no malate was detected over the whole course of the experiment, both the T-strain as well as the PMT-strain produced significant amounts of malate. After 72 h the PMT- and T-strain still produced comparable amounts of 4.1 ± 0.2 g/L and 3.4 ± 0.1 g/L malate, respectively (Fig. 3A). However, after that, production stopped in the T-strain and a final titer of only 3.6 ± 0.0 g/L malate was reached at the end of the cultivation. Production in the PMT-strain still continued and reached a malate concentration of 7.7 ± 0.4 g/L after 144 h. These results highlight that the transporter enabling malate export out of the cells is essential to enable high-level malate production. It is also striking that the PMT-strain, although showing high malate production, showed nearly no growth when cultured with methanol as carbon source (Fig. 3B). A possible explanation could be the high activity of NADH shuttle systems, such as the malate-aspartate shuttle, under these growth conditions. The malate-aspartate shuttle translocates electrons across the mitochondrial membrane to make them available for energy generation via the respiratory chain [36] (Fig. 3C). For the methylotrophic yeast *Komagataella phaffii* (*Pichia pastoris*), it has been reported that the malate-aspartate shuttle is highly active when methanol is present in the medium [37], which reflects the high maintenance energy associated with growth on methanol compared to growth on glucose [38, 39]. Consequently, it can be expected that in *O. polymorpha* growth on methanol triggers a high flux towards malate. When a highly efficient exporter such as Mae1(p) transports this malate from the cytosol into the extracellular space, this would prevent the transport of electrons into the mitochondria and thus limit ATP generation via the respiratory chain. Hence, this could be a possible cause for the growth defect observed after the introduction of the transporter into *O. polymorpha*, and it could at the same time explain why overexpression of the transporter gene alone already leads to an increase in malate production.

**Fig. 4:**
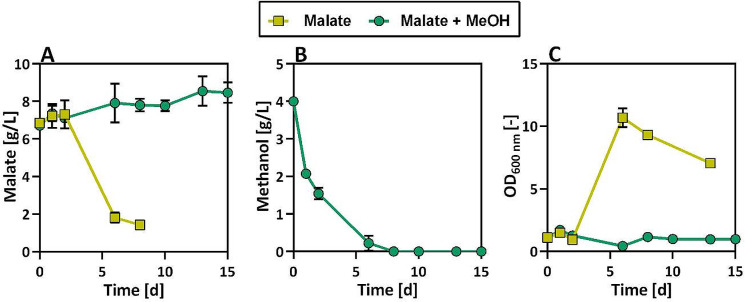
Malate uptake of *O. polymorpha* PMT-strain. Cultivation in Verduyn medium with either 6.7 g/L malate (green circles) or 6.7 g/L malate and 4 g/L methanol (yellow squares) as carbon source. Measurement of malate (**A**) and methanol (**B**) concentration and optical density (**C**). Cultivation was performed in System Duetz microtiter plates. Error bars represent the standard deviation of three biological replicates

As the PMT-strain showed promising results overall, this strain was further characterized. It was evaluated whether the PMT-strain could take up malate from the medium. Hence, the PMT-strain was cultivated in medium with 6.7 g/L (50 mM) of externally supplied malate as well as a mixture of 6.7 g/L malate and 4 g/L methanol (Fig. 4).

**Fig. 5:**
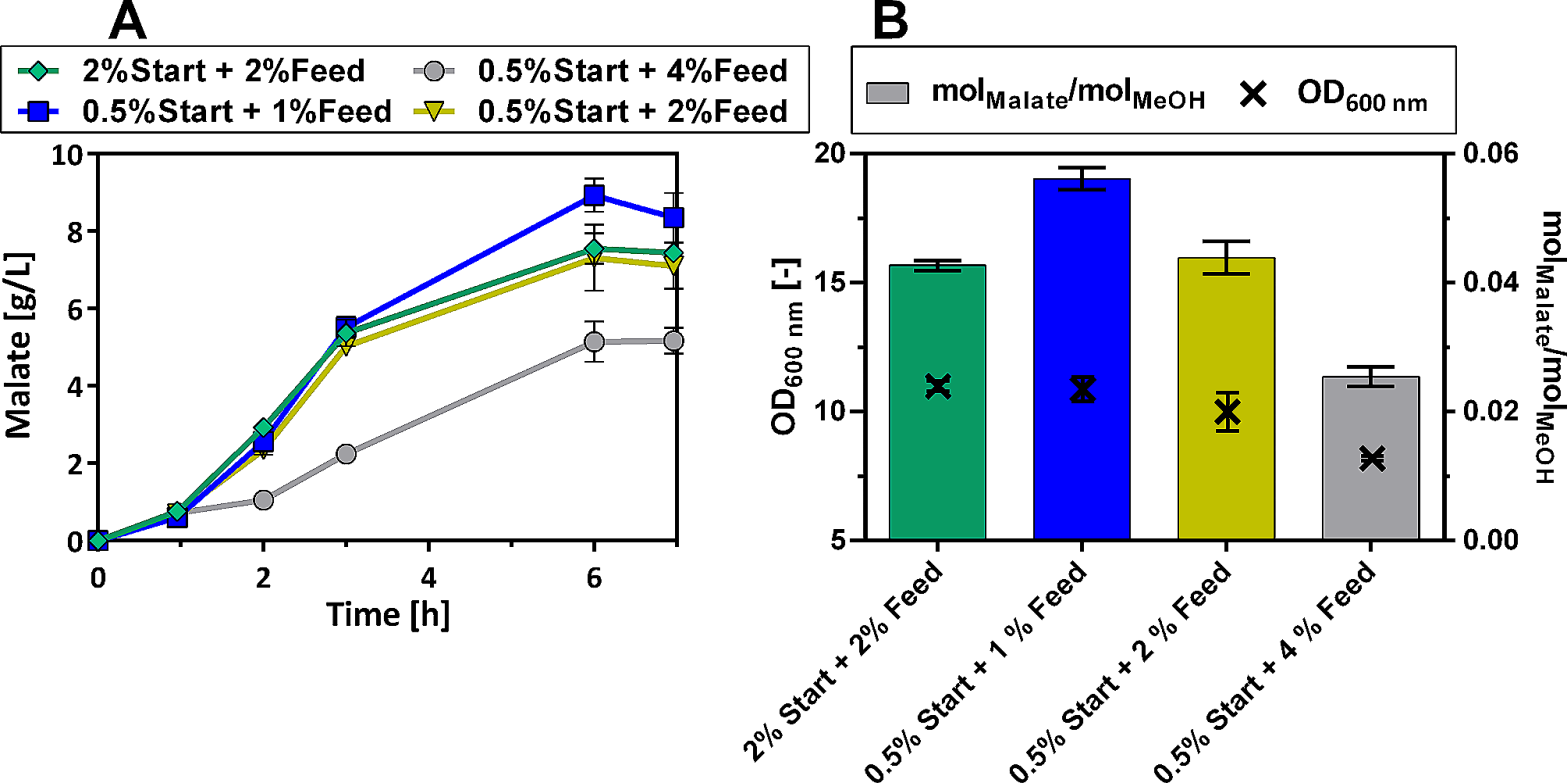
Analyzing the effect of different feeding patterns on malate production. (**(A)**)Measurement of malate concentration throughout the experiment. (**B**)Optical density at the end of the experiment and amount of malate produced per amount of consumed methanol after 6 days (in mol_Malate_/mol_MeOH_). The experiment was performed in 250 mL shake flasks on Verduyn medium with either 0.5% or 2% of initial methanol concentration and then feeding pulses of 1%, 2%, or 4% once the methanol concentration was depleted. Error bars represent the standard deviation of the biological replicates

When malate was supplied to the medium as the sole available carbon source, the cells were able to take up the malate and grow to an optical density of 10.7 ± 0.7 within five days (Fig. 4C). In contrast to that, the cells did not consume malate, when methanol was used as an additional carbon source, but instead consumed the methanol (Fig. 4A&B). Here, it can be observed again that the used PMT-strain takes up methanol as a substrate but does not channel this assimilated carbon into biomass formation as there is no growth on methanol observed (Fig. 4C). This experiment shows how methanol seems to be a preferred carbon source compared to malate and highlights how in future experiments it will be essential to make methanol available at all time points of the cultivation to avoid the uptake of malate by the yeast. Consequently, different methanol feeding strategies were tested. It has to be considered that the expression of all heterologous genes in the malate-producing strain is controlled through methanol-inducible promoters. Therefore, different inducing methanol concentrations of 0.5% (4 g/L) and 2% (16 g/L) were tested. Once these initial methanol concentrations were nearly depleted, additional methanol pulses between 1% and 4% were added to the cells. With these varied cultivation modes, the experiments were carried out for 7 days (Fig. 5).

Evaluating these results, it can be concluded that feeding the strain frequently with low methanol concentrations is beneficial for production. The highest malate titer of 8.9 ± 0.4 g/L was achieved, when the strain was supplied with an initial methanol concentration of 0.5% methanol and was then fed with a fresh pulse of 1% methanol every day (Fig. 5). In contrast to that, feeding the strain with pulses of 4% methanol led to a comparably low maximal titer of 5.7 ± 0.3 g/L, which is likely related to the toxicity of methanol at higher concentrations.

Also, when considering malate production in relation to the amount of methanol consumed, higher yields were achieved by feeding low concentrations of methanol to the medium. While feeding pulses of 1% methanol led to a yield of around 0.056 mol_Malate_/mol_MeOH_, the cells that were fed with 4% only achieved a 45% lower yield of 0.025 mol_Malate_/mol_MeOH_. Therefore, lower methanol concentrations of 1% were used for feeding the cultures in the following experiments.

**Fig. 6:**
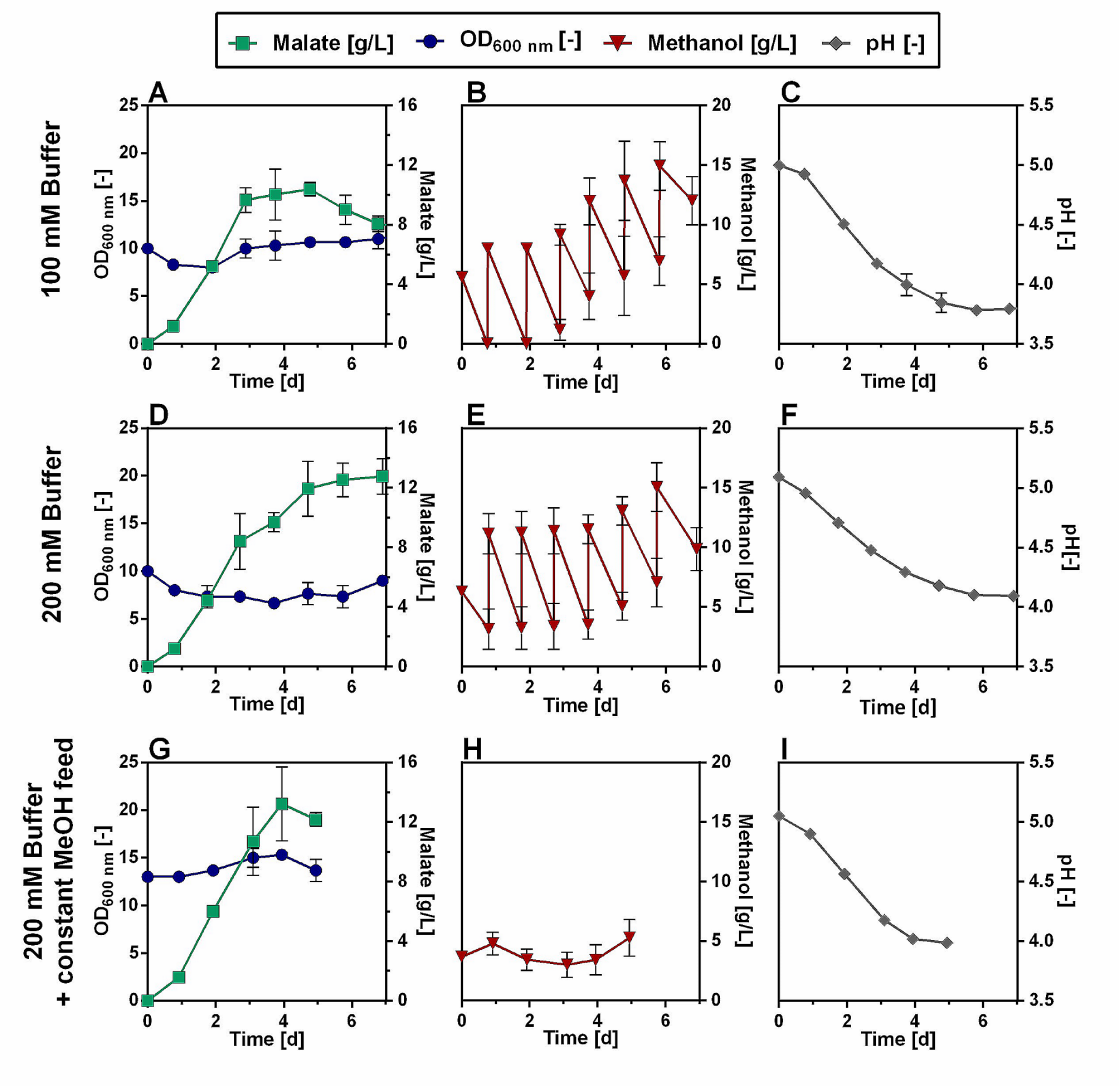
Malate production of *O. polymorpha* PMT-strain in methanol fed-batch experiment in shake flasks. Malate production (**A**, **D**, green squares), optical density (**A, D**, blue circles), methanol concentration (**B, E**, red triangles), and pH (**C, F**, grey diamonds) in shake flasks that were fed with 8 g/L (1%) of methanol every 24 h. Comparison of Verduyn medium with 100 mM of KH-phthalate (**A, B, C**) and 200 mM KH-phthalate (**D, E, F**) as buffer. Panels(**G, H & I**) represent an experiment where shake flasks were fed with a constant methanol feed using the LIS system. (**G**: OD (blue circles), Malate concentration (green squares), **H**: Methanol concentration, **I**: pH)

To get a more accurate idea of when and under what conditions malate is produced in *O. polymorpha*, the PMT-strain was fed with 1% methanol daily for one week. Each day, the malate and methanol concentrations and the pH in the medium were measured (Fig. 6A-C).

It was observed that the rate of malate production was highest during the first three days of cultivation. During this time the cells produced 9.7 ± 0.8 g/L of malate (Fig. 6A). After that, production slowed down considerably and a maximum titer of 10.4 ± 0.5 g/L was obtained on day 5 of the cultivation. After that, the malate titers decreased slightly. Some byproducts were observed as well during this cultivation. Succinate was the main byproduct, which can be explained by the fact that the transporter Mae1(p) can also transport other dicarboxylic acids apart from malate [34]. At the time when the highest malate titer was measured (day 5), the corresponding succinate titer reached 0.5 ± 0.0 g/L (SI: Figure S3). As the malate titer decreased in the following days, the succinate titer continued to increase. In addition to succinate, minor amounts of pyruvate (max. titer 38 mg/L) and fumarate (max. titer 7 mg/L) were also detected during this cultivation. It is noticeable that the by-products are initially produced at a low rate and then the production rate increases when malate production stops after day 3. Consequently, we expected that with the optimization of malate production in this strain, the production of byproducts will decrease. During the first two days of the cultivation, the supplied methanol pulses of 1% are still completely consumed from the medium within one day. However, over time an accumulation of methanol in the culture occurs. At the end of the cultivation, the final methanol titer reached 375 ± 63 mM (Fig. 6B). At the same time, it was observed that the pH dropped from an initial value of 5.0 to a value of 3.8 (Fig. 6C). This was assumed to be a major reason, why the production of malate stopped at one point during the experiment. According to a study by Camarasa et al. (2001), the transporter Mae1(p) is reliant on the pH difference between the cytoplasm and extracellular space [40]. In heterologous expression studies of *MAE1* in *Saccharomyces cerevisiae* it was observed that malic acid import is restricted and export is encouraged at the pH level of the medium above 4. In comparison, a lower pH enables the cell to import malic acid [40]. This corresponds well to the observed phenomenon in this experiment, where the malate titer decreases as soon as the pH drops below a level of 4 (Fig. 6A&C). Further, it has to be considered that with a decrease in pH, a higher percentage of the acid will be present in its protonated form (pK_a1 Malic acid_: 3.4). The protonated acid can thus diffuse more easily through the cell membrane and lead to an acidification of the cytoplasm [41]. This might be an additional reason, why malate production stops after day 3. Consequently, controlling the pH of the cultivation medium will be a critical factor for improving malate production. As a first step to do so, the amount of buffer used in the cultivation medium was doubled from a concentration of 100 mM KH-phthalate (Fig. 6A-C) to 200 mM (Fig. 6D-F). Increasing the buffer concentration led to a slower drop in the pH and during the whole cultivation, the pH remained at a level above 4.0 (Fig. 6F). In comparison with using 100 mM of buffer, malate production also improved. The malate titer in the culture increased over the whole course of the experiment and reached a final value of 12.7 ± 1.2 g/L (Fig. 6D) after 7 days. At the same time, methanol accumulation happened more slowly in this cultivation with a final methanol titer of only 9.8 ± 1.8 g/L at the end of the cultivation. This experiment highlights that control of pH is a critical parameter for malate production in this strain and that a pH value above 4 seems to be advantageous for production.

Moreover, it was assumed that keeping the methanol concentration during the cultivation constant could improve production. The chosen method of manual addition of methanol pulses in shake flasks, although technically simple and straightforward, does not allow accurate feeding and leads to fluctuating methanol concentrations in the culture (Fig. 6B&E). This could stress the cells by alternating between carbon starvation and methanol toxicity. Therefore, the Liquid Injection System (LIS, SBI Scientific Bioprocessing, Baesweiler, Germany) was tested, which allows constant substrate feeding to shake flasks. With this system, a total daily concentration of 1% of methanol was supplied in a constant feed to the cultures (Fig. 6G-I). Like this, the methanol concentration in the cultures was successfully kept constant (Fig. 6H), which also improved malate production. After 4 days a titer of 13.2 ± 2.5 g/L malate was obtained (Fig. 6G). In the previous experiment, without constant methanol feeding (Fig. 6D-F), a similar maximum titer of 12.7 ± 1.2 g/L was achieved, but it took seven days to reach this titer. The constant methanol feeding thus considerably decreased the necessary cultivation time. During the first 4 days of the cultivation malate is thus produced at a rate of 3.4 g/L/d Additionally, the production of byproducts decreased during this cultivation. At the timepoint where the highest malate titer was measured 0.1 ± 0.0 g/L succinate was detected (SI: Figure S3). This is only 20% of what has been observed under the same conditions with only 100 mM buffer and manual methanol feeding. Again, it can clearly be seen that as soon as the pH reaches a value around 4.0 production stops and malate is even taken up. This emphasizes how it will also be essential to further optimize pH levels to improve malate production in this strain. Still, the methanol titers and yields that are already achieved with the non-optimized cultivation strategy in this study are highly promising. So far, the only reported instance in which malate was produced using methanol as a substrate is in *Pichia pastoris*, another methylotrophic yeast. Guo et al. reported the production of 2.8 g/L of malic acid from methanol with an engineered *P. pastoris* strain [30]. Additionally, it should be considered that the malate titers obtained in this study were achieved without the addition of neutralizing agents such as CaCO_3_, which are often added during production processes of organic acids to avoid acid stress and thus boost production. Although the addition of CaCO_3_ for neutralization may have the potential to further increase malate production in *O. polymorpha*, it also makes purification of the acid produced more laborious [42], so the costs and benefits of this approach should be carefully assessed in subsequent experiments.

Further optimizing this cultivation in a more controlled setting, e.g., in a bioreactor that allows both pH control and constant methanol feeding will be of critical importance to further improve malate production in *O. polymorpha*. As bioreactor cultivations further allow for the optimization of oxygen supply, it will also be an interesting option to exploit *O. polymorpha’s* suitability for high cell density fermentations, which could push malate production even further.

### Acetone production

**Fig. 7:**
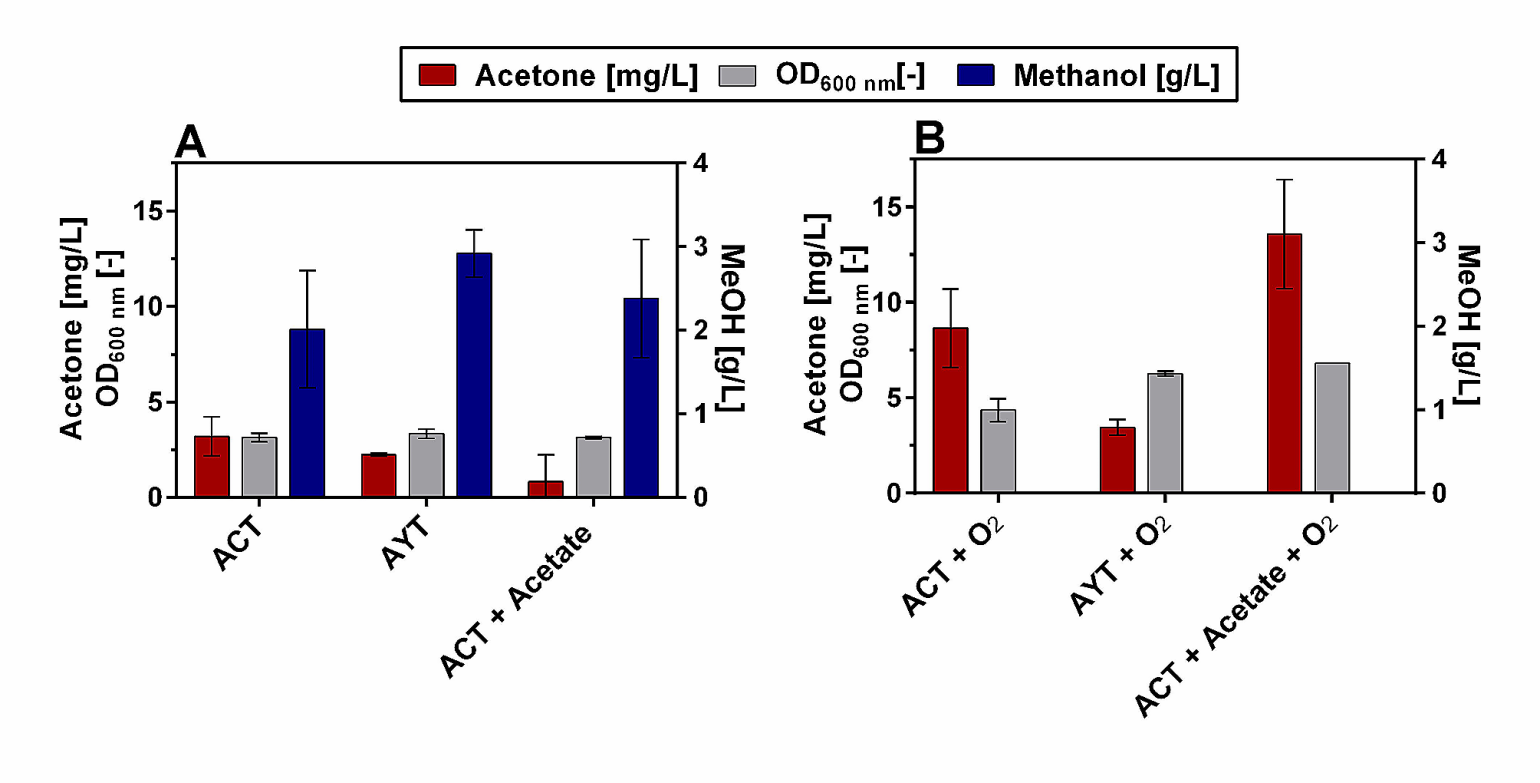
Acetone titer, final optical density, and methanol concentration for *O. polymorpha* strains overexpressing heterologous genes for acetone synthesis. *O. polymorpha* strains either overproducing PpAdc(p), CtfAB(p), and ThlA(p) (ACT) or PpAdc(p), YbgC(p), and ThlA(p) (AYT). Strains were cultivated in 250 mL air-tight serum bottles with 25 mL Verduyn medium + 4 g/L (0.5% (v/v)) methanol. For the ACT strain, an additional condition was analyzed where 1.5 g/L acetate were added to the medium (+ Acetate). The headspace consisted either of 100% air (**A**) or of 50% air and 50% pure oxygen (**B**). Error bars represent the standard deviation of biological triplicates

To enable acetone synthesis in *O. polymorpha* the precursor acetyl-CoA has to be converted in three enzymatic steps to acetone via aceto-acetyl-CoA and acetoacetone as intermediates. In *Clostridium acetobutylicum* these reactions are catalyzed by an acetyl-CoA acetyltransferase (thiolase, ThlA(p), EC 2.3.1.9), a butyrate-acetoacetate CoA-transferase (CtfAB(p), EC 2.8.3.9) and subsequently by an acetoacetate decarboxylase (Adc(p), EC 4.1.1.4) (Fig. 1). Therefore, this study aimed to introduce variants of all these three enzymes into *O. polymorpha* to enable acetone synthesis. For the first enzymatic step catalyzed by the acetyl-CoA acetyltransferase, the *THLA* gene from *Clostridium acetobutylicum* was chosen. For the intermediate reaction step, there was also a clostridial gene variant (*CTFAB*) selected. However, CtfAB(p) requires acetate as a cofactor and at the same time has a high Km value for acetate (1200 mM). This is often considered to be the limiting factor in heterologous acetone production [43]. As an alternative to CtfAB(p) the YbgC(p) enzyme from *Haemophilus influenzae* was also introduced into *O. polymorpha*. YbgC(p) is a thioesterase (EC:3.1.2.-) that allows acetate-independent hydrolysis of acetoacetyl-CoA to form acetoacetate and has already been applied in *Escherichia coli* for heterologous acetone production [44]. For the last reaction step instead of the clostridial variant (Adc(p)), an alternative enzyme from *Paenibacillus polymyxa* (*Pp*Adc(p)) was introduced into *O. polymorpha*. This enzyme is reported to have a comparable turnover number and a more favorable Km value for acetoacetone than the clostridial variant (Specific activity: 269 µmol/min/mg; Km 0.94 mM [45]). Consequently, two *O.polymorpha* strains were engineered that overproduced either *Pp*Adc(p), CtfAB(p) and ThlA(p) (ACT-strain) or *Pp*Adc(p), YbgC(p) and ThlA(p) (AYT-strain). All of the heterologous genes were codon-optimized for *O. polymorpha* expressed using either the strong methanol-inducible MOX or CAT promoter (SI Table S1). To allow the detection of the volatile product acetone, the two created strains were cultivated in 250 mL air-tight serum bottles using 0.5% methanol as carbon source. Additionally, a supplementation of 25 mM ammonium acetate to the medium was tested for the ACT-strain, as the CtfAB(p) requires acetate as a cofactor. After 5 days of cultivation, the cultures were sampled. The final optical density as well as the concentration of acetone and leftover methanol in the cultivation medium were determined (Fig. 7A).

For both the ACT and AYT strain, acetone production was demonstrated successfully. These strains produced 3.2 ± 1.0 mg/L (ACT) and 2.2 ± 0.1 mg/L acetone (AYT), respectively (Fig. 7A). Adding acetate to the medium of the ACT strain did not increase acetone production. For all strains, the 0.5% methanol supplied in the medium were not consumed completely. For the AYT strain 91.2 ± 8.8 mM of methanol remained in the culture broth, which corresponds to 73% of the initially supplied methanol concentration. As the used serum bottles are completely air-tight to avoid evaporation of the produced acetone the only oxygen available for the cells to grow is the headspace in the culture. As growth on methanol is strictly aerobic in *O. polymorpha*, it was assumed that the yeast cells could not consume the entire methanol due to oxygen being limiting. Therefore, the experiment was repeated, but this time 50% of the headspace was replaced with pure oxygen (Fig. 7B). With these experimental conditions, all cultures completely consumed methanol from the cultivation medium, which also led to an increase in the final optical density. At the same time, acetone titers increased. The AYT strain produced 3.5 ± 0.4 mg/L acetone, while the ACT strain even reached a titer of 8.6 ± 2.1 mg/L. Adding acetate to the medium even pushed acetone production further in the ACT strain to 13.6 ± 2.9 mg/L. The precursors for acetone are also part of *O. polymorpha’s* native metabolism. There is, e.g., a native isozyme of the acetyl-CoA acetyltransferase (EC.2.3.1.9; *ERG10*) as part of the mevalonate pathway (MVA pathway) in *O. polymorpha*. The mevalonate pathway is essential for ergosterol synthesis and thus for growth. We can therefore assume that there will be production of acetoacetyl-CoA under a variety of different culture conditions. Therefore, it was tested whether the introduction of *Pp*Adc(p) alone or *Pp*Adc(p) in combination with CtfAB(p) or YbgC(p) already resulted in acetone production. However, there was no acetone detected in any of these created strains (data not shown). Consequently, of the tested strains the ACT strain with supplemented acetate in the medium and oxygen-enriched headspace produced the highest acetone titer in this study. These acetone titers are far from what can be achieved in an ABE fermentation with *Clostridium acetobutylicum* as a native acetone producer [46]. However, to our knowledge, this is the first time that a heterologous production pathway for acetone was successfully introduced in a yeast as a heterologous host. Even though titers are still low these results provide a proof-of-principle that acetone production from methanol as a substrate is possible.

### Production of isoprene

To enable isoprene synthesis in *O. polymorpha* an isoprene synthase (Isps(p), EC:4.2.3.27) has to be introduced, that can catalyze the formation of isoprene from dimethylallyl diphosphate (DMAPP) as a substrate. DMAPP is a native intermediate of the mevalonate pathway in yeast species. In this study, two different variants of the isoprene synthase were tested, one from *Pueraria montana* (kudzu vine) and the other one from *Populus alba* (white poplar). Both of these genes have already been applied successfully for the heterologous production of isoprene in different microbial species [28, 47, 48]. As isoprene synthase genes typically have low turnover numbers and high Km values [49], it has been reported, that increasing the number of copies of isoprene synthase genes enhances the isoprene production [50]. Therefore, we also created *O. polymorpha* strains with a double integration of the respective isoprene synthase cassette. Further, we aimed at increasing the supply of the precursor DMAPP as it has been described extensively, how to increase the flux through the mevalonate pathway in yeast species [18, 51, 52]. The irreversible conversion of 3-hydroxy-3-methylglutaryl coenzyme A (HMG-CoA) to mevalonate, catalyzed by the enzyme 3-hydroxy-3-methylglutaryl-CoA reductase (Hmgr(p)) is regarded as the initial rate-limiting step of the mevalonate pathway [53, 54]. In yeasts, this enzyme has an N-terminal signal peptide that anchors it to the endoplasmic reticulum membrane. Deleting this anchor and thus making the enzyme available in the cytosol, has been described as one of the key strategies to enable a higher flux through the mevalonate pathway [55]. Thus, a truncated copy of the *HMGR* gene (*tHMGR*) from *Saccharomyces cerevisiae* was additionally introduced into the genome of *O. polymorpha* and overexpressed using the strong methanol-inducible MOX promoter.

**Fig. 8:**
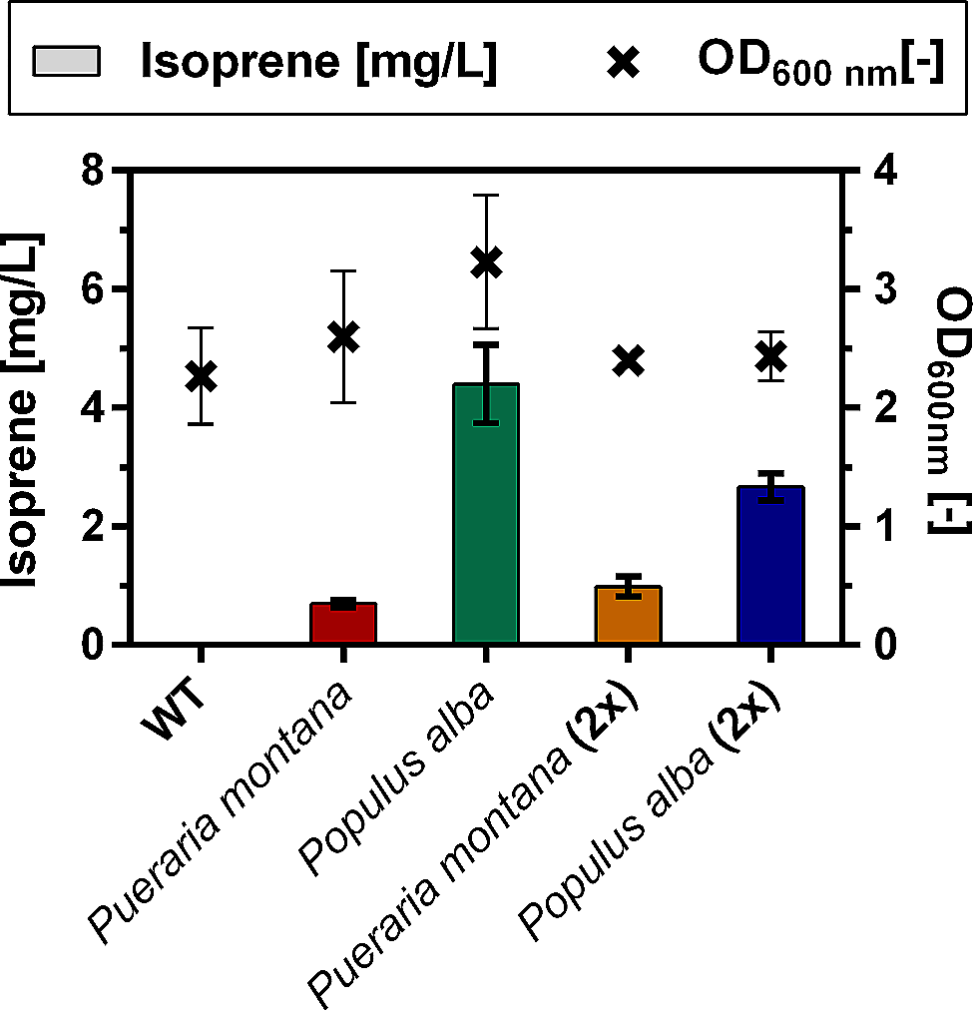
Production of isoprene from methanol in *O. polymorpha* strains overproducing isoprene synthases (Isps(p)) from either *Pueraria montana* or *Populus alba*. In strains marked with “(2x)”, the respective isoprene synthase gene was integrated twice. The unmodified wildtype (WT) strain was included as a negative control. Error bars represent standard deviations of the biological replicates

The created *O. polymorpha* strains were subsequently transferred to closed serum bottles on Verduyn medium containing 0.5% MeOH as the sole carbon source and cultivated at 37 °C. Consistent with the experiments with the acetone-producing strains, 50% of the headspace of the culture was replaced with pure oxygen. As isoprene has a boiling point of 34 °C, isoprene was quantified from the headspace of the culture. As a control the unmodified *O. polymorpha* wildtype (WT) strain was included in the experiment. Figure 8 shows the amount of produced isoprene in these experiments.

While the unmodified WT strain did not produce any isoprene, all other strains showed isoprene production. The strain expressing a single copy of the *Populus alba ISPS* gene showed the highest isoprene production of 4.4 ± 0.7 mg per L of culture medium. The strain expressing the single copy of the *Pueraria montana ISPS* gene produced considerably less isoprene compared to the *Populus alba* strain (0.7 ± 0.1 mg/L). It was also observed that the strain overexpressing the *Populus alba* isoprene synthase gene grew to a higher biomass density than the WT strain. This might be linked to the expression of the isoprene synthase gene pulling more carbon flux into the mevalonate pathway, which also provides the precursors for ergosterol synthesis, that are essential for growth [56]. It was further assessed whether an increased gene copy number could further enhance isoprene production, by introducing a second copy of the respective isoprene synthase gene into these strains. However, introducing another copy of the *Pueraria montana ISPS* gene only led to a slightly increased isoprene titer of 1.0 ± 0.2 mg/L. Surprisingly, the introduction of a second copy of the *Populus alba* isoprene synthase even led to a decreased titer compared to the single copy integration strain (2.7 ± 0.2 mg/L). As the isoprene synthase genes are under the control of strong methanol-inducible promoters (SI: Table S1), this decreased production might suggest an increased metabolic burden resulting from the elevated gene expression. This would also be reflected in the fact that the strain expressing a second copy of the isoprene synthase of *Populus alba* no longer showed improved biomass formation compared to the unmodified WT strain. Since the single integration strain of the *Populus alba* ispS produced most isoprene, further cultivation conditions were tested for this strain. As the *Populus alba* isoprene synthase has its optimal reaction temperature synthase at 40 °C [57], isoprene production of the *Populus alba* strain was analyzed at an elevated cultivation temperature. However, this elevated temperature led both to a decrease in growth and isoprene production (SI: Figure S4). This could be related to the increased volatility of the substrate methanol at higher temperatures, whereby less methanol is available in the cultivation medium and growth and product formation are correspondingly impeded. Further, as isoprene synthases require magnesium as a cofactor, we assessed whether an increased magnesium content in the medium might improve production. The magnesium titer in the medium was increased to 20 mM a concentration at which the *Populus alba* isoprene synthase was reported to be activated [57]. With this medium adaptation however, isoprene production stayed at the same level (4.59 ± 0.09 mg/L) as with the original medium, indicating that the availability of magnesium is not a limiting factor in the cultivation (SI: Figure S4).

The highest isoprene titer obtained in this study (4.4 mg/L) is already substantially higher than the titers that were achieved with similarly modified strains of *Saccharomyces cerevisiae* (0.5 mg/L, [50]) and *Yarrowia lipolytica* (0.53 mg/L, [47]) cultivated on glucose. This highlights the potential of *O. polymorpha* as a possible isoprene production organism. Compared to other yeasts, *O. polymorpha* as a thermotolerant organism further has the advantage that it can be cultivated at a temperature above the boiling point of isoprene. In addition to avoiding product inhibition and potentially facilitating product purification, this has the advantage that the cultivation temperature is closer to the temperature optimum of the isoprene synthases, potentially allowing the enzymes to function more efficiently than in mesophilic organisms. In the model organism *S. cerevisiae*, isoprene production from glucose has already been further advanced by extensive genetic modifications and optimization of cultivation conditions, which ultimately enabled isoprene production at a gram-per-liter scale. These optimization strategies include targeting an additional copy of the isoprene production pathway to the mitochondria [58], directed evolution of the isoprene synthase [59], and performing fed-batch cultivations at high cell densities [60]. These strategies indicate potential ways to also increase isoprene production from methanol in *O. polymorpha* in the future.

### Perspectives for producing methanol-derived chemicals in *O. polymorpha*

In this study, we successfully demonstrated the production of malate, acetone, and isoprene using methanol as carbon source for the yeast *O. polymorpha*. Malate could be produced with highly promising titers of 13.2 g/L and productivities of 3.4 g/L/d. This reflects that *O. polymorpha* appears to be an excellent organism for malate production, possibly due to the high activity of the malate-aspartate shuttle when methanol is used as a carbon source. These results, therefore, also underline the suitability of methylotrophic yeasts for producing carboxylic acids from C1 molecules, as similar titers can neither be achieved with photoautotrophic bacteria growing on CO_2_ nor with methylotrophic bacteria at the moment [61–63]. Still, studies reporting the production of low molecular weight biochemicals in methylotrophic yeasts using methanol as substrate are currently rare in the literature. This might be related to the intrinsic properties of methanol metabolism in yeast. The majority of the assimilated carbon is oxidized to CO_2_ in the dissimilatory branch of methanol metabolism. When methanol is used as a carbon source, 50–70% of the total carbon flux in the methylotrophic yeast *P. pastoris* passes through this dissimilatory branch [64]. As a result, metabolism during growth on methanol is characterized by low carbon flux to pyruvate and even lower flux to acetyl-CoA (33% and 13% of the methanol uptake flux, respectively) [65]. While this means that there are few overflow metabolites produced during growth on methanol, it makes the production of chemicals derived from these precursors challenging. This is also reflected in the fact that the production of acetone and isoprene with *O. polymorpha* in this study only occurs at comparatively low levels and that the yield of malate on methanol only reaches 0.099 mol/mol, which corresponds to 40% of the theortical maximum yield. To enable the synthesis of larger amounts of methanol-derived chemicals in yeasts, it will be crucial to reroute the carbon flux towards precursors such as pyruvate and acetyl-CoA in addition to optimizing the respective biosynthesis pathways. Potential strategies that have been suggested for this include the overexpression of the dihydroxyacetone synthase (DHAS) to increase the assimilation of formaldehyde [66], knocking out the glucose-6-phosphate isomerase to reduce flux through the pentose phosphate pathway [30] or downregulation of the dissimilatory branch of methanol assimilation [38, 65]. All these strategies bear the risk of disturbing the delicate balance in methanol metabolism and leading to an accumulation of toxic intermediates such as formaldehyde and thus have to be carefully tested.

Apart from optimizing the precursor supply, developing strategies to efficiently avoid methanol toxicity will be essential for successfully establishing the production of platform chemicals from methanol. In this study, utilization of the LIS system allowed constant methanol feeding to shake flasks and was demonstrated to be a simple solution to circumvent substrate toxicity already during the early characterization phase of newly constructed strains, a technology that can be used beyond methanol and yeast.

## Conclusion

This study successfully demonstrates the production of three different industrially relevant chemicals in the yeast *Ogataea polymorpha* using methanol as a substrate. It highlights how the yeast’s metabolism is not equally well suited for the production of all of these molecules and thus also emphasizes the necessity for further engineering of the methanol metabolism of methylotrophic yeast. These findings serve as a point of reference for future metabolic engineering efforts of *O. polymorpha* to boost the production of low molecular weight compounds from methanol.

## Methods

### Strains and media

The strains engineered in this study are based on the strain *Ogataea polymorpha* NCYC495 *leu1.1* ∆yku80 [67]. Pre-cultures of *O. polymorpha* were grown at 37 °C in YPD medium (10 g/L yeast extract, 20 g/L peptone, 20 g/L glucose). If not otherwise stated, the defined mineral Verduyn medium [68] buffered with 100 mM KH-Phthalate and variable concentrations of methanol as carbon source, was used for all cultivations. In addition, the medium was supplemented with 0.5 g/L leucine, as the constructed strains are leucine-auxotrophic, and with 1 g/L yeast extract as an additional nitrogen source and auxiliary substrate, which reportedly improves methanol utilization [30]. A detailed description of the medium composition can be found in the supplementary information (SI: Table S2-4). For plasmid propagation, competent *Escherichia coli* cells (NEB® 10-beta, High Efficiency) were used. *E. coli* cells were grown at 37 °C in LB medium, supplemented as required with 100 µg/L ampicillin.

### Strain construction

The overexpressed genes in this study were codon-optimized for *O. polymorpha* and ordered as synthetic DNA fragments via the GeneArt gene synthesis service (Thermo Fisher Scientific, Waltham, MA, USA). As an exception, the truncated 3-hydroxy-3-methylglutaryl-CoA reductase gene (*tHMGR*) was directly amplified from the genome of *Saccharomyces cerevisiae*. All overexpressed genes were integrated into the genome of *O. polymorpha* using the pHIP expression vectors [67]. The created pHIP expression plasmids are listed in the Supplementary Information (SI: Table S1). For the expression of the CtfAB(p) enzyme from *Clostridium acetobutylicum*, the genes for both the A and B subunit were co-expressed in *O. polymorpha* using the ERBV-1 (Equine rhinitis B virus) 2 A peptide [69].

For integration, the pHIP vectors were linearized at unique restriction sites in the promoter region of the vector and then transformed as linear cassettes into *O. polymorpha* through LiAc/single-stranded carrier DNA/PEG transformation [70]. For selection after transformation, the cells were spread on YPD agar plates supplemented with either 200 µg/mL hygromycin, 100 µg/L zeocin, or 100 mg/L nourseothricin.

### Cultivation conditions

The malate-producing *O. polymorpha strains* were cultivated in 250 mL shake flasks or 24-well System Duetz microtiter plates, filled with 25 mL or 3 mL Verduyn medium, respectively. Cultivations were performed at 37 °C with 250 rpm shaking. For the constant methanol feeding experiment, the Liquid Injection System (LIS, SBI Scientific Bioprocessing, Baesweiler, Germany) was used, which allows automated feeding of liquids into shake flask cultures. During cultivation, the shake flasks were weighed daily to determine the evaporation rate of the medium. The measured product titers and optical densities were then corrected for this evaporation.

The strains producing acetone or isoprene were cultivated in 250 mL serum bottles which were sealed with air-tight rubber stoppers, to prevent evaporation of the volatile products during cultivation. The bottles were filled with 25 mL cultivation medium. To avoid oxygen limitation of the cells, 50% of the headspace was removed from the flask and replaced with pure oxygen.

### Analytical methods

High-performance liquid chromatography (HPLC-UV-RI) was used to monitor methanol consumption and malate production. To this end, 1 mL of yeast culture was centrifuged at maximum speed and the supernatant was filtered through Rotilabo syringe filters (Carl Roth, pore size 0.2 m). The filtered supernatants were then analyzed using a DIONEX UltiMate 3000 HPLC System (Thermo Fisher Scientific) equipped with a Metab-AAC column (300 × 7.8 mm, ISERA, Düren, Germany). 5 mM H_2_SO_4_ was used as running buffer at a flow rate of 0.6 mL/min and a temperature of 30 °C. A SHODEX RI-101 detector (Showa Denko Europe GmbH, München, Germany) and a DIONEX UltiMate 3000 Variable Wavelength Detector (Thermo Fisher Scientific) set to 210 nm were used for detection. Analytes were identified by comparison of the retention time and UV/RI quotient to standards.

For acetone quantification, the serum bottles used for cultivation were cooled to 4 °C, after which samples were taken from the culture broth, filtered through Rotilabo syringe filters (Carl Roth, pore size 0.2 μm) and then directly stored in screw-cap GC-vials at 4 °C. A Trace GC Ultra GC (Thermo Fisher Scientific) equipped with a flame ionization detector (FID) was used for the determination of acetone titers. Analytes were separated with a Zebron ZB-WAX column (length: 30 m, film thickness: 0.25 μm, inner diameter: 0.25 mm, Zebron, Phenomenex, UK). The measurement was performed with helium as carrier gas with a flow rate of 2 mL/min, an injection volume of 1 µL, a split ratio of 1:40, an inlet temperature of 220 °C, and the following temperature profile: 40 °C constant for 2.5 min, an increase of 35 °C/min to 150 °C, an increase of 40 °C/min to 250 °C, 250 °C constant for 3 min.

For isoprene analytics, 1 mL of culture headspace was taken from the culture with a Hamilton 1001 gas-tight syringe (Hamilton Bonaduz AG, Bonaduz, Switzerland). The headspace samples were analyzed with an Agilent 8890 gas chromatograph with a flame ionization detector (FID) (Agilent Technologies Inc., Santa Clara, CA, USA), equipped with a Pora BOND Q column (50 m, Inner diameter: 0.32 mm, film thickness: 5 μm, Agilent Technologies Inc.). Helium was used as carrier gas at a flow rate of 1.8 mL/min, and the following temperature profile was applied: 50 °C constant for 5 min, an increase of 8 °C/min to 250 °C, 250 °C constant for 15 min.

### Simulations

We adapted the existing genome scale metabolic model of *O. polymorpha* (iUL909) to arrive at the new model iOpol23 (https://github.com/iAMB-RWTH-Aachen/Opol-GSMM). The new model contains reactions for isoprene synthase (EC: 4.2.3.27), acetoacetyl-CoA hydrolase (EC: 3.1.2.11), acetoacetate decarboxylase (EC: 4.1.1.4) and transport reactions for acetone and isoprene. The yield simulations were performed with methanol as sole carbon source with an uptake rate of 10 mmol/gDW/h. Different scenarios of metabolic fluxes were tested for their capacity to generate the products and Table 2 shows the respective settings of the genome scale model. The boundaries for AKGDam, ICL, and MDHm were manually identified as the minimum/maximum fluxes that still supported feasible solutions. There are in total 15 combinations of simulations (3 target metabolites in 5 pathway scenarios) and all flux solutions are provided as supplementary material. The flux balance analysis simulations were performed with cobrapy (v0.17.1) in Python (v3.9.7) with the standard optimization function [71]. The principal component analysis (PCA) was conducted with scikitlearn (v1.0.2). A Jupyter Notebook with a workflow of the whole analysis is available on GitHub (https://github.com/iAMB-RWTH-Aachen/Opol-GSMM).

**Table 2:** Settings of the GSMM iOpol23 to simulate five different pathway scenarios. The setting of the ‘Unconstraint Reference’ is also the basis for the remaining four scenarios

Scenario	Model Adaptation
Unconstraint Reference (Ref)	MeOH rate: 10 mmol/gDW/h Objective: malate/acetone/isoprene
Blocked TCA (TCA-)	= Ref and: Upper bound = 0 for: (PDHa1, PDHcm, ACLSm, AKGDH1 + 2, CITtam, CITtap, CSp + m)
Forced TCA (TCA+)	= Ref and: Lower bound = 7.8 for: AKGDam
Forced Glyoxylate shunt (Glx + Std)	= Ref and: Lower bound = 10 for: ICL
Forced, Impaired Glyoxylate shunt (Glx + Mut)	= Glx + Std and: Lower/Upper bound = -10/2.85 for: MDHm

### Electronic supplementary material

Below is the link to the electronic supplementary material.

Supplementary Material 1

Supplementary Material 2

## Data Availability

The data produced in this study are presented in the article or additional material. More accessible and interactive data is available at: https://github.com/iAMB-RWTH-Aachen/Opol-GSMM. This repository contains an Excel-file with the experimental data for Figs. 3, 4, 5, 6, 7 and 8, the adapted genome scale metabolic model of *O. polymorpha* (iOpol23), and a Jupyter Notebook with a workflow of the simulation.
