# Supplementary material for: Methanol bioconversion into C3, C4, and C5 platform chemicals by the yeast Ogataea polymorpha
Source: Microb Cell Fact. 2024 Jan 3;23:8. doi: 10.1186/s12934-023-02283-z (PMC10763331; doi:10.1186/s12934-023-02283-z)
Supplement: Supplementary file 2 — Supplementary Material 2 [file 12934_2023_2283_MOESM2_ESM.pdf]

Supplementary Information

Methanol bioconversion into C3, C4 and C5 platform chemicals by the yeast *Ogataea polymorpha*

Katrin Wefelmeier[1], Simone Schmitz[1], Benjamin Jonas Kösters[1], Ulf Winfried Liebal[1], Lars Mathias Blank[1*]

1 iAMB - Institute of Applied Microbiology, ABBt – Aachen Biology and Biotechnology, RWTH Aachen University, Worringerweg 1, D-52074 Aachen, Germany

** Correspondence: lars.blank@rwth-aachen.de*

Used plasmids

***Table S1**: Plasmids*

Plasmid Name	Backbone	Features (promoter_GOI_terminator)	Selectable Marker	Restriction site used for linearization	Origin
pHIPH4	-	pMOX/tAMO	Hygromycin resistance	-	Saraya et al., 2011
pHIPN4	-	pMOX/tAMO	Nourseothricin resistance	-	Saraya et al., 2011
pHIPZ15	-	pDHAS/tAMO	Zeocin resistance	-	See: https://www.rug.nl/research/molecular-cell-biology/research/the-hansenula-polymorpha-expression-system?lang=en
pKW5	-	pCAT_ubiGFP_tMOX	Zeocin resistance	-	Wefelmeier et al. 2022
SZ10	-	pMOX_lacZ_tMOX	Hygromycin resistance	-	Wefelmeier et al. 2022
#0836	pKW5	pCAT_Pa-ispS_tMOX	Zeocin resistance	SpeI	This study
#0837	pKW5	pCAT_Pm-ispS_tMOX	Zeocin resistance	SpeI	This study
#0838	pHIPN4	pDHAS_Pm-ispS_tMOX	Nourseothricin resistance	HindIII	This study

#0839	pHIPN4	pDHAS_Pa-ispS_tMOX	Nourseothricin resistance	EcoRV	This study
MAE1_NourR	pHIPN4	pDHAS_MAE_tENO	Nourseothricin resistance	BstBI	This study
#0842	pKW5	pCAT_Ro-MDH_tMOX	Zeocin resistance	SpeI	This study
#0843	SZ10	pMOX_Ro-PYC_tMOX	Hygromycin resistance	SphI	This study
pKW32	pHIPH4	pMOX_YbgC_tAMO	Zeocin resistance	NsiI	This study
pKW33	pHIPH4	pMOX_CTFA_2A_CTFB_tAMO	Zeocin resistance	NsiI	This study
pKW40	SZ10	pMOX_Pp-ADC_tMOX	Hygromycin resistance	SphI	This study
pKW42	pHIPN4	pCAT_thlA_tAMO	Nourseothricin resistance	BamHI	This study

Verduyn medium composition

Table S2*: Composition of mineral Verduyn medium*

Component	Concentration
$(NH_4)_2SO_4$	5 g/L
KH_2PO_4	3 g/L
$MgSO_4 \cdot 7H_2O$	0.5 g/L
1000X Vitamin solution	1 mL/L
100X Trace elements	10 mL/L
L-Leucine	0.5 g/L
Yeast extract	1 g/L
KH-Phthalate	100 mM
• adjust pH to 5 with 5 M KOH<br>• supplement with desired concentration of methanol as carbon source	

Table S3: Composition of 1000X vitamin solution for Verduyn medium

Component	Concentration
D-biotin	0.05 g/L
Ca-D-pantothenate	1.00 g/L
Nicotinic acid	1.00 g/L
Myo-inositol	25.00 g/L
Thiamine hydrochloride	1.00 g/L
Pyridoxal hydrochloride	1.00 g/L
p-aminobenzoic acid	0.20 g/L

Table S4: Composition of 100X trace elements solution for Verduyn medium

Components	Composition
Na_2EDTA	1.50 g/L
$ZnSO_4 \cdot 7H_2O$	0.45 g/L
$MnCl_2 \cdot 2H_2O$	0.10 g/L
$CoCl_2 \cdot 6H_2O$	0.03 g/L
$CuSO_4 \cdot 5H_2O$	0.03 g/L
$Na_2MoO_4 \cdot 2H_2O$	0.04 g/L
$CaCl_2 \cdot 2H_2O$	0.45 g/L
$FeSO_4 \cdot 7H_2O$	0.30 g/L
H_3BO_3	0.10 g/L
KI	0.01 g /L

Overview of constraint reactions

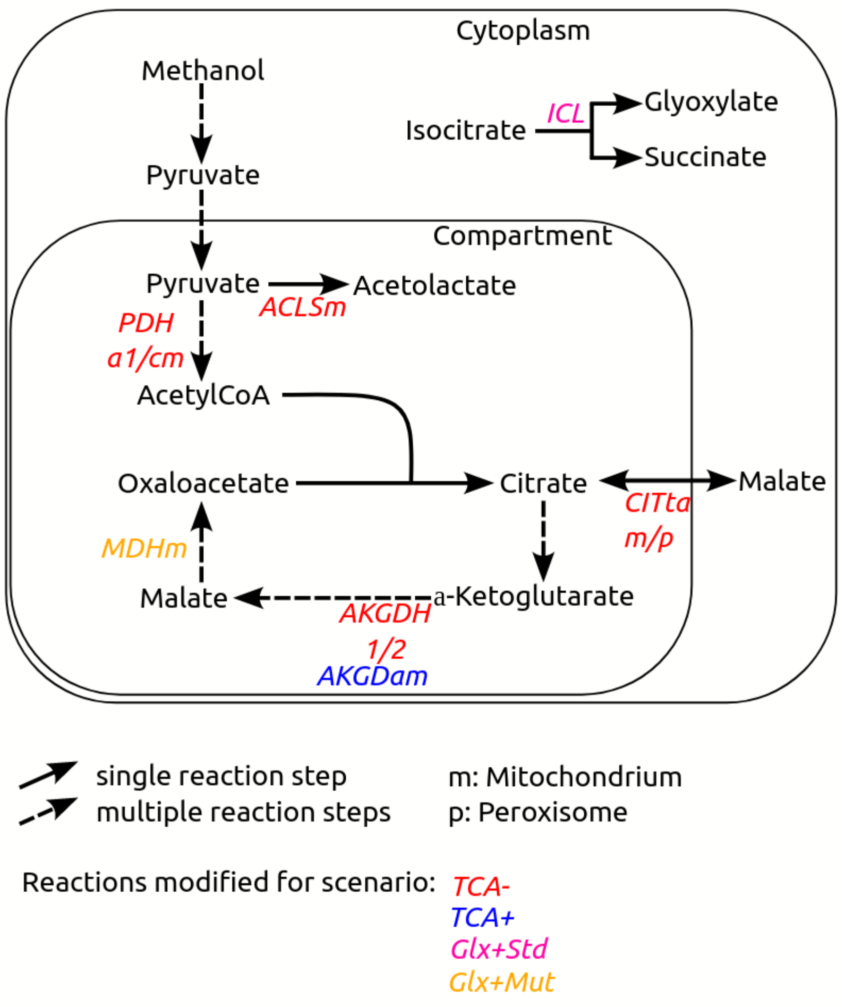

Figure S 1 *- Provides an overview to the reactions that have been constraint to define the networks of the different scenarios TCA-, TCA+, Glx+Std, Glx+Mut.*

Methanol consumption

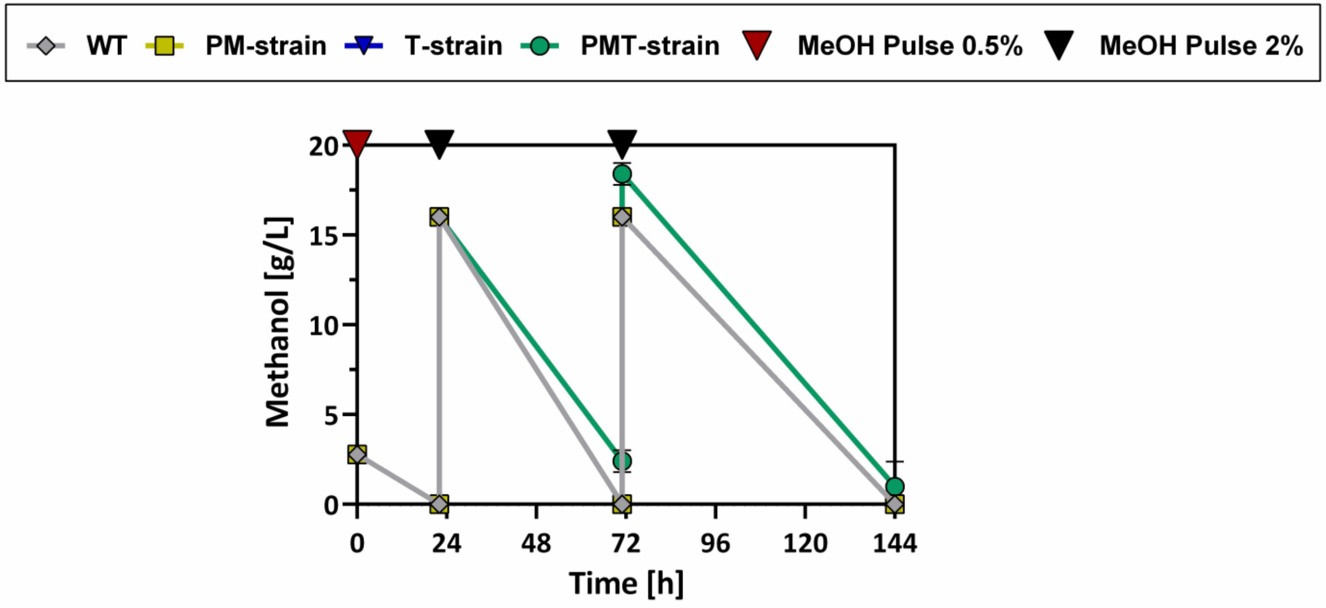

***Figure S 2** – Comparison of methanol consumption between malate producing* O. polymorpha *strains. Comparison of the unmodified wildtype (WT) strain, a strain overexpressing the* PYC *and* MDH *gene of* Rhizopus oryzae *(PM), a strain overexpressing the malate transporter* MAE1 *of* Schizosaccharomyces pombe *(T) and a strain overexpressing all three of these heterologous genes (PMT). Cultivations were performed on Verduyn medium in 250 mL shake flasks. Error bars represent the standard deviation of biological triplicates.*

Byproduct formation during malate production

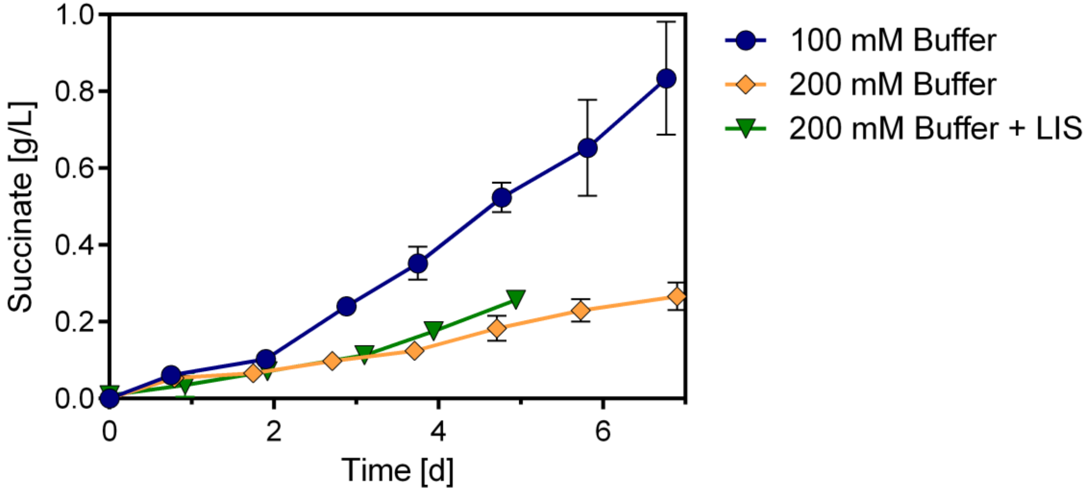

***Figure S 3** – Succinate formation of malate producing* O. polymorpha *PMT-strain in methanol fed-batch experiment in shake flasks Comparison of Verduyn medium with 100 mM of KH-phthalate (blue circles) and 200 mM KH-phthalate (yellow diamonds) as buffer in shake flasks that were fed with 1% (v/v) of methanol every 24 h. In a third condition shake flasks were fed with a constant methanol feed of 1% pure methanol/24 h using the Liquid Injection System (green triangles).*

Adapted cultivation conditions for isoprene production

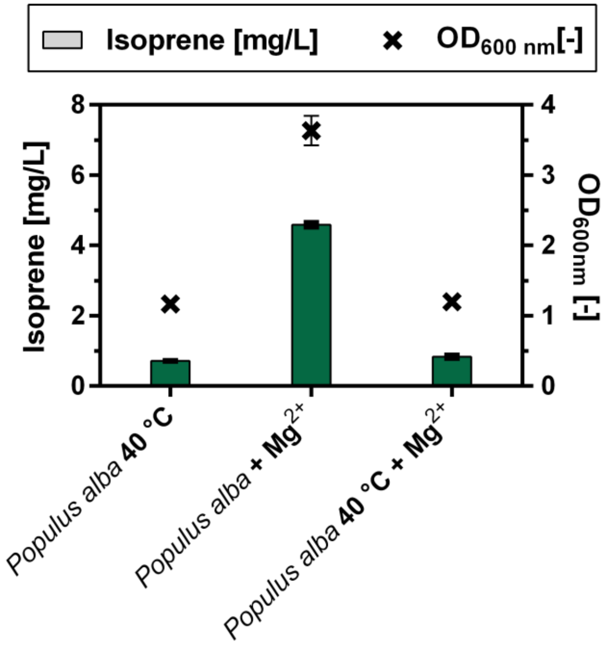

Figure S 4 *- Production of isoprene from methanol in* O. polymorpha *strains overproducing a single copy of the isoprene synthase of* Populus alba. *The strain was cultivated with and elevated temperature of 40 °C and with an increased magnesium concentration (+ Mg^{2+}) in the medium and a combination of both (40 °C + Mg^{2+}). Error bars represent standard deviations of the biological replicates.*

References

Saraya, R., Krikken, A. M., Kiel, J. A., Baerends, R. J., Veenhuis, M., & van der Klei, I. J. (2012). Novel genetic tools for Hansenula polymorpha. *FEMS yeast research*, *12*(3), 271-278.

Wefelmeier, K., Ebert, B. E., Blank, L. M., & Schmitz, S. (2022). Mix and match: promoters and terminators for tuning gene expression in the methylotrophic yeast Ogataea polymorpha. *Frontiers in Bioengineering and Biotechnology*, *10*, 876316.
